# Classification of breast cancer using a manta-ray foraging optimized transfer learning framework

**DOI:** 10.7717/peerj-cs.1054

**Published:** 2022-08-08

**Authors:** Nadiah A. Baghdadi, Amer Malki, Hossam Magdy Balaha, Yousry AbdulAzeem, Mahmoud Badawy, Mostafa Elhosseini

**Affiliations:** 1 College of Nursing, Nursing Management and Education Department, Princess Nourah bint Abdulrahman University, Riyadh, Saudi Arabia; 2 College of Computer Science and Engineering, Taibah University, Yanbu, Saudi Arabia; 3 Computers and Control Systems Engineering Department, Faculty of Engineering, Mansoura University, Mansoura, Egypt; 4 Computer Engineering Department, Misr Higher Institute for Engineering and Technology, Mansoura, Egypt

**Keywords:** Breast cancer, Convolutional neural network (CNN), Deep learning (DL), Metaheuristic optimization, Manta-Ray foraging algorithm (MRFO)

## Abstract

Due to its high prevalence and wide dissemination, breast cancer is a particularly dangerous disease. Breast cancer survival chances can be improved by early detection and diagnosis. For medical image analyzers, diagnosing is tough, time-consuming, routine, and repetitive. Medical image analysis could be a useful method for detecting such a disease. Recently, artificial intelligence technology has been utilized to help radiologists identify breast cancer more rapidly and reliably. Convolutional neural networks, among other technologies, are promising medical image recognition and classification tools. This study proposes a framework for automatic and reliable breast cancer classification based on histological and ultrasound data. The system is built on CNN and employs transfer learning technology and metaheuristic optimization. The Manta Ray Foraging Optimization (MRFO) approach is deployed to improve the framework’s adaptability. Using the Breast Cancer Dataset (two classes) and the Breast Ultrasound Dataset (three-classes), eight modern pre-trained CNN architectures are examined to apply the transfer learning technique. The framework uses MRFO to improve the performance of CNN architectures by optimizing their hyperparameters. Extensive experiments have recorded performance parameters, including accuracy, AUC, precision, F1-score, sensitivity, dice, recall, IoU, and cosine similarity. The proposed framework scored 97.73% on histopathological data and 99.01% on ultrasound data in terms of accuracy. The experimental results show that the proposed framework is superior to other state-of-the-art approaches in the literature review.

## Introduction

With over two million cases diagnosed globally in 2020 and about seven hundred thousand deaths, breast cancer is classified as the most lethal female disease (Sung et al., 2021). By 2040, the number of patients is anticipated to rise to 4.07 million, with 1.4 million deaths (Global Cancer Observatory, 2020). With approximately 26% of women affected worldwide, it is regarded as the first cancer type to cause death. However, detecting and diagnosing breast cancer in its early stages can enhance survival rates by up to 80% (Yu et al., 2022). Clinicians must identify suspicious tumors after segmenting them to diagnose breast abnormalities. As the number of breast cancer patients climbs, clinicians will find it increasingly difficult to accurately detect the disease in a short time (Oza et al., 2022). For medical image analysts, the diagnosis process is complex, time-consuming, and monotonous. Medical image analysis is one of the most exciting research areas that have gained significant attention in academia and the medical sector, allowing for the detection and treatment of various illnesses, including breast cancer (Dewangan et al., 2022; Zerouaoui & Idri, 2022; Chowdhury et al., 2022; Mohamed et al., 2022). Breast cancers are classified into five stages according to Peiris et al. (2015). The stages progress from pre-cancerous to metastatic or advanced cancer. Digital mammography is a tool for identifying early-stage breast cancer with no need for surgery (Demb et al., 2020). With mammography screening, radiologists can detect cancers in mammogram images with smaller diameters and seemingly random places. The ability of mammography to detect significant early signs has resulted in a 15% reduction in breast cancer deaths (Løberg et al., 2015). Breast ultrasound is a regularly used diagnostic modality in clinical practice (Pourasad et al., 2021). Breast cancer is caused by epithelial cells surrounding the terminal duct lobular unit. Cancer cells inside the basement membrane of the draining duct and the basement membrane of the terminal duct lobular unit are referred to as *in situ*, or non-invasive cancer cells (Jabeen et al., 2022). The presence of axillary lymph node metastases is an important factor when selecting prospective therapy for breast cancer (Sun et al., 2020). Ultrasound imaging is one of the most commonly used test materials for detecting and characterizing breast disorders. It is a common imaging method for mammography and radiological cancer diagnostics.

Artificial intelligence (AI) technology has lately been used to aid radiologists in detecting breast cancer more quickly and accurately utilizing mammography (Zhang et al., 2021). Radiologists may concentrate on discrete or localized areas, but AI-based systems analyze mammograms as a whole at the pixel level and have spatially long-range memory. AI strategies for enhancing mammographic detection of breast cancer have been widely studied, involving several issues and challenges. The use of artificial intelligence (AI) in mammography analysis is widely thought to make the computer-aided diagnosis (CAD) process more interesting, effective, and objective. Earlier CAD systems relied on handcrafted features to recognize suspicious bulk using traditional machine learning algorithms (Muramatsu et al., 2016; Virmani et al., 2016; Yassin et al., 2018; Li et al., 2017). Manual feature extraction has led to a higher number of false positives (Dhungel, Carneiro & Bradley, 2017). Because the image has numerous details, identifying the optimal set of features for recognition becomes quite difficult. Deep learning methods have overcome the shortcomings of traditional machine learning approaches by learning an object’s attributes during the training phase (Rupapara et al., 2022; Gardezi et al., 2019).

In recent decades, convolution neural networks (CNN) have been used in CAD systems for breast cancer diagnosis. It functions as a decision-making mechanism by giving extra information to discriminate between malignant and benign tumors (Oza et al., 2022). The CAD can potentially increase the overall accuracy, sensitivity, and specificity of breast lesion diagnosis. In addition, this approach cuts down the number of false positives caused by human mistakes. Several researches that discuss the use of CAD systems to diagnose breast cancer have been published in the literature, some of which have already passed clinical testing (Yu et al., 2022; Bruno et al., 2020). This study introduces a framework for reliable breast cancer classification based on histopathological and ultrasound data using CNN and Transfer Learning (TL) (Agarwal et al., 2021). The Manta Ray Foraging Optimization (MRFO) algorithm is used for parameters and hyperparameters optimization. The following points outline the current study’s contributions:

– Presenting a novel CNN-based framework for automatic and reliable breast cancer classification based on histopathological and ultrasound data.
– Applying Transfer Learning using eight modern pre-trained models.
– Optimizing CNN and Transfer Learning hyperparameters *via* MRFO algorithm to achieve the best configurations for each pre-trained model and better classification performance.
– The hyperparameters of the CNN architecture do not need to be set manually, making the proposed framework more adaptable.
– The results reveal that the worthiness of the proposed framework exceeds most related research in terms of accuracy and other criteria.

The rest of the article is organized as follows: In ‘Background’, the background of deep learning, CNN, and metaheuristic optimization are introduced. ‘Related Studies’ reviews the related studies of breast cancer classification. In ‘Methodology’, the approach is described, as well as the proposed framework. In ‘Numerical Results and Analysis’, the experimental results are examined and debated. Finally, in ‘Conclusions’, the article is concluded.

## Background

This section presents comprehensive preliminaries for the most relevant subjects and methodologies employed in this study. Convolutional neural network (CNN), transfer learning (together with the pre-trained CNN networks utilized in this study), metaheuristic optimization, and the MRFO optimization technique are among the subjects covered.

### Convolutional neural network (CNN)

**Figure 1:**
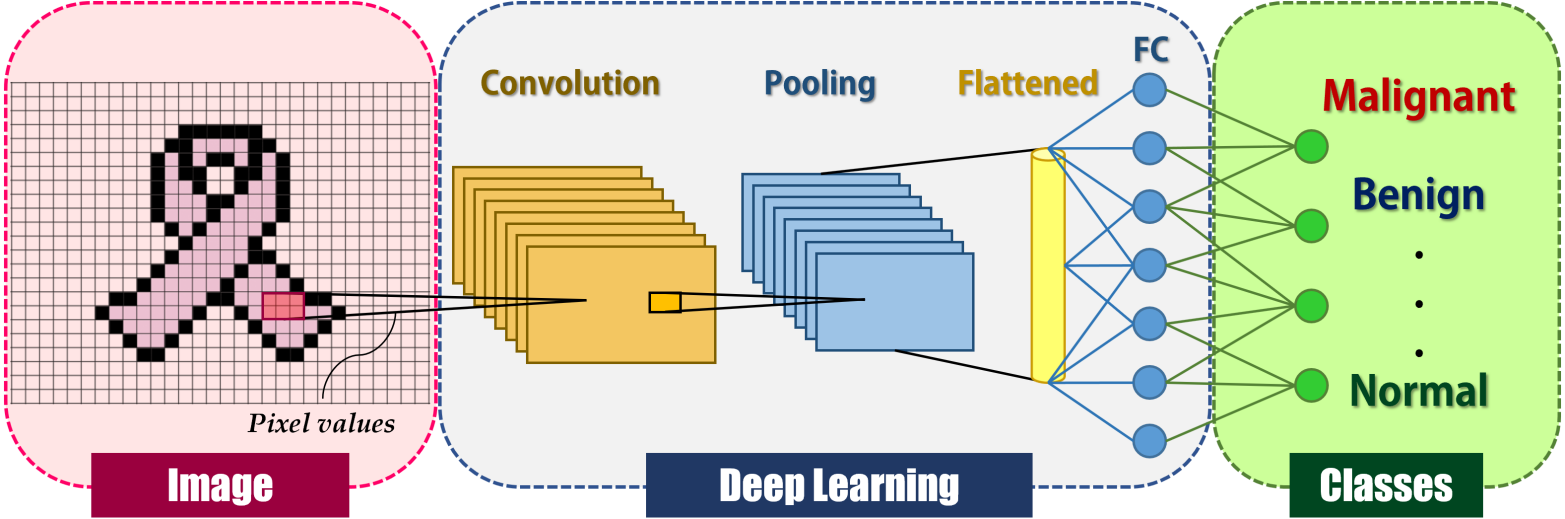
General deep learning architecture for image classification.

Hinton invented the term “deep learning” in 2007 to describe machine learning models for high-level object representation, although it was not widely acknowledged until late 2012 (LeCun, Bengio & Hinton, 2015). Deep learning gained prominence in the field of computer vision after a deep learning technique based on a convolutional neural network (CNN) won the most well-known computer vision competition, ImageNet (Krizhevsky, Sutskever & Hinton, 2012). CNN is a deep learning system inspired by the human brain’s visual cortex and seeks to mimic human visual function (Sarvamangala & Kulkarni, 2021). CNN is a significant advancement in image comprehension, including image classification, segmentation, localization, and detection. The fundamental reason for CNNs’ extensive adoption is their efficiency in image recognition. The CNN takes an image as input and produces classification categories such as cancer or non-cancer. Local shift-invariant interconnections connect the layers. CNN is a multi-layer generative graphical model that employs pixel values in images as input information rather than features generated from segmented objects, obviating the need for feature calculation or object segmentation. CNNs, like human neurons, are made up of convolutions with learnable weights and biases. Convolutional layers, activation functions, pooling, and fully-connected layers are the primary building blocks of CNNs, as depicted in Fig. 1.

### Transfer learning

Training and testing data should be in the same feature space, according to a key idea in many machine learning (ML) algorithms. This hypothesis, however, may not hold true in various real-world applications. For example, there are instances where training data is prohibitively expensive or difficult to obtain (Baghdadi et al., 2022). As a result, high-performance classifiers trained using increasingly available data from different domains are required. This strategy is known as transfer learning (TL) (Agarwal et al., 2021). When the target domain has limited data, but the source domain has a large dataset, TL performs brilliantly. The process of obtaining information from a source domain DS that contains a learning task TS and applying that knowledge to enhance a learning task TT is informally described as TL. In a target domain, DT, where DS is not the same as DT or TS is not the same as TT (Zhuang et al., 2020; Iman, Rasheed & Arabnia, 2022).

#### CNN architectures

This section highlights the CNN architectures pre-trained to perform transfer learning. Among several designs, InceptionV3, Xception, EfficientNetB7, NASNetLarge, VGG19, SeNet154, DenseNet201, and ResNet152V2 are discussed since they were used in this work.

##### InceptionV3:

With transfer learning, InceptionV3 achieved high classification accuracy in numerous biomedical applications (Ashwath Rao, Kini & Nostas, 2022; Al Husaini et al., 2022). The symmetric and asymmetric construction components of the InceptionV3 model include convolutions, average pooling, max pooling, concatenations, dropouts, and fully connected layers (Szegedy et al., 2016). Batch normalization is employed frequently throughout the model and for activation inputs. In addition, InceptionV3 employs Softmax to compute Loss, Max-pooling for spatial pooling, and FC for final classification.

##### Xception:

Xception, which stands for Extreme version of Inception, was proposed as an expansion of the Inception architecture that was fully built on depth-wise different convolutions rather than conventional convolutions (Chollet, 2017). The use of depth-wise distinct convolutional layers in the CNN network simplifies network computations. This aids in learning the efficient features from images and improves model performance.

##### EfficientNetB7:

The core building component of EfficientNet architecture is Mobile Inverted Bottleneck Convolution (MBConv) (Sandler et al., 2018). The number of these MBConv chunks varies within the EfficientNet network family. From EfficientNetB0 through EfficientNetB7, depth, breadth, resolution, and model size increase, as does accuracy. In terms of ImageNet accuracy, EfficientNetB7, outperforms prior state-of-the-art CNNs (Tan & Le, 2019). Parameters like filter size, stride, and channel count divide the network architecture of EfficientNetB7 into seven blocks. As a result, EfficientNet is a popular image classification and segmentation tool.

##### NASNetLarge:

The neural architecture search techniques develop the Neural Architecture Search Network Large (NASNet-Large) (Zhang, Wu & Zhu, 2018). It comprises two types of layers or cells: Normal Cells and Reduction Cells. The Reduction Cell decreases the width and height of the feature map along the forward route, whereas the Normal Cell keeps these two dimensions the same as the input feature map. Each Normal/Reduction Cell is made up of several blocks. Each block is constructed from a series of popular deep CNN operations with varying kernel sizes.

##### VGG19:

The VGG-19 deep learning algorithm is a 16-layer network with three FC layers (Simonyan & Zisserman, 2014). Maxpool, FC, Relu, dropout, and softmax layers are among the 41 total layers. VGG tackled the large-size filter impact by replacing it with a stack of (3*times*3) filters. However, the calculation complexity is increased by using small-size filters.

##### SeNet154:

Squeeze-and-Excite Networks provide a building element for CNNs that improves channel interdependencies at nearly no computational cost (Hu, Shen & Sun, 2018). Aside from providing a significant speed improvement, they are also simple to include in current systems. The Squeeze and Excitation Network, in essence, proposes a novel channel-wise attention mechanism for CNNs in order to improve their channel interdependencies. They learn to use global information, boosting advantageous features while ignoring others.

##### DenseNet201:

Dense convolution network (DenseNet) is a pre-trained deep learning model that employs feedforward to connect each layer to all subsequent levels (Huang et al., 2017). A feature map is included in each layer of the model. The feature map of each layer serves as the input for the following layer. It allows optimum information transfer inside the network by connecting all levels directly. DenseNet’s key benefits are that it drastically reduces the number of parameters, reduces gradient runaway, improves feature diffusion, and encourages feature reuse.

##### ResNet152V2:

A Residual Network (ResNet) is a CNN architecture composed of several convolutional layers. Previous CNN configurations reduced additional layers’ efficiency. As a result, ResNet has many layers and is highly performant (He et al., 2016). The main difference between ResNetV2 and the previous (V1) is that, before adding a weight layer, V2 performs batch normalization on it. Consequently, ResNet performs well in image recognition, illustrating the significance of a wide range of visual recognition tasks.

### Metaheuristic optimization

Optimization is a critical procedure for making the best use of various resources. It is a necessary step in any field of study. Several strategies have been developed to address various optimization challenges (Meraihi et al., 2021). Each algorithm employs a particular acceptable technique for certain problems and efficiently solves them while being ineffective for others (Abualigah et al., 2022). However, the vast majority of them fall into one of two groups. The conventional approaches, such as gradient descent and Newton (Wang et al., 2021), fall under the first category. These approaches are basic and straightforward in general, but they only yield a single solution every iteration, which is time-consuming. The strategies in the second category, known as metaheuristic (MH), tend to escape the limits of traditional methods (Alshinwan et al., 2021). Nowadays, MH optimization methods are highly appealing due to their particular benefits over traditional algorithms. MH is used to identify high-quality solutions to a wider range of challenging real-world scenarios since it can handle multi-objective multi-modal, and nonlinear formulations. These MH strategies are generally inspired by nature, physics principles, and human behavior. The primary classes of MH include natural phenomena-based, swarm-based, human-based, and evolutionary-based techniques (Abualigah et al., 2022). Natural phenomena-based approaches imitate natural phenomena such as spirals, rain, wind, and light (Ewees et al., 2021; Şahin, Dinler & Abualigah, 2021). The swarm-based methods mimic the behavior of animals, birds, fish, and other swarms when they are looking for food (Sharma & Kaur, 2021; Zhao, Zhang & Wang, 2020). As optimization methods, human-based methods mimic human behavior (Moosavi & Bardsiri, 2019; Khishe & Mosavi, 2020). Finally, the mechanism of evolutionary-based approaches is inspired by emulating the notions of natural genetics, which is employed to replicate the principle of natural genetics (Tzanetos & Dounias, 2021; Bentéjac, Csörgo & Martnez-Muñoz, 2021). These approaches rely on the operators’ crossover, mutation, and natural selection.

#### Manta ray foraging optimization

Manta Ray Foraging Optimization (MRFO) (Zhao, Zhang & Wang, 2020) is a swarm MH optimization algorithm. It was inspired by the manta rays’ foraging activity when acquiring their food. Chain foraging, cyclone foraging, and somersault foraging are three strong and sophisticated foraging tactics evolved by manta rays. Chain foraging emulates the fundamental activity of food hunting. The hunting manta rays form a chain to capture all the plankton on the way. Manta rays consume plankton, which is regarded as a food resource. The chain leader of the manta ray guides the chain as it has the best solution. This process decreases the risk of losing plankton and increases food hunted. When a high plankton density is located, The manta rays swim in a spiral pattern towards the plankton during cyclone foraging. The spiral route is determined by the location of plankton and the relative position of a manta ray in relation to its front agent. This mechanism produces a vertex in the eye of a cyclone, allowing manta rays to capture the plankton easily. Somersault foraging starts by considering the plankton’s best position as a pivot. Then, the searching manta ray performs backward somersaults before rolling around the pivot. Later on, the leader, manta ray, updates its location with the best location so far. Despite their uncommon nature, these foraging activities are exceedingly effective.

## Related studies

Most breast cancer detection strategies have relied on machine learning or deep learning to classify cases into binary or multi-class classifications. Accuracy, precision, recall, and F1-score performance indicators are all tracked using these methodologies. Breast cancer diagnosis and categorization have recently become the subject of substantial investigation. Wang et al. (2016) proposed a deep learning-based approach for automatically detecting metastatic cancer using entire slide images of sentinel lymph nodes. To improve the training set, they included patches from normal lymph node regions that the system had initially misclassified as cancer. The method used a 27-layer deep network architecture and achieved 98.40% classification accuracy. Han et al. (2017b) developed a deep learning-based breast cancer multi-classification approach. The structured model worked brilliantly on a large-scale dataset (averaging 93.2% accuracy). This indicates the proposed strategy’s strength in terms of providing a useful tool for breast cancer multi-classification. Han et al. (2017a) employed a deep learning framework to distinguish between different types of tumors in breast ultrasound imaging. The training data used ten-fold cross-validation to find the optimal values. The networks had a 91% accuracy rate.

Jannesari et al. (2018) used pre-trained deep neural networks and fine-tuned them to distinguish between several cancer types such as breast, bladder, lung, and lymphoma. This method was then applied to the BreakHis database to categorize breast cancer subtypes. They were able to perform breast tumor binary classification with 96.4% accuracy. Golatkar, Anand & Sethi (2018) created a two-stage system for categorizing tissue images into four groups: normal, benign, *in situ*, and invasive. First, they employed a pre-processing technique to select only important regions from tissue images for training and testing. Next, they employed transfer learning to train the patch-level classifier and majority voting to classify images. The average accuracy across the four classifications was 85%, with carcinoma *vs.* non-carcinoma classification accuracy of 93%. Fujioka et al. (2019) employed a CNN to classify breast mass images obtained from ultrasound. The CNN model had an accuracy of 92.5%. Compared to radiologists, the CNN model performed better in the binary classification of breast tumors based on ultrasound.

Hijab et al. (2019) investigated three deep learning variations for ultrasound (US) images for computer-aided recognition of breast cancers. To improve accuracy, they fine-tuned and employed the VGG16 pre-trained model. The accuracy of the results ranges from 79 to 97%. Singh et al. (2020) embraced the notion of employing transfer learning in breast cancer, where they worked on unbalanced data from the WSI dataset and employed VGG-19 with other classifiers such as logistic regression, random forest, and other dense layers. As a result, they were able to achieve maximum accuracy of 90.30%. In their study, Ayana, Dese & Choe (2021) focused on transfer learning methods applied to ultrasound breast image classification and detection utilizing pre-trained CNN models. Furthermore, their examination of some of the most regularly utilized transfer learning approaches highlights the potential for future study in this area. In their research, Khamparia et al. (2021) proposed a hybrid transfer learning model incorporating MVGG and ImageNet. They used the WSI dataset to test their model, and its accuracy was 94.3%. They also employed image segmentation and 3D mammography throughout their study, which helped them get superior results.

Choudhary et al. (2021) conducted extensive experiments with three significant pre-trained CNNs, including VGG19, ResNet34, and ResNet50. They attained an accuracy of 91.25% using the VGG19 pruned model, outperforming early techniques on the same dataset. Alzubaidi et al. (2021) proposed a solution to the data scarcity problem in medical imaging. The authors employed many unlabeled histopathological images of breast cancer to train the CNN model. The model was fine-tuned before being trained on a small labeled breast cancer dataset. The authors attained a 97.51% overall accuracy with this method. The authors also used new double transfer learning, which resulted in a 97.7% accuracy. Dewangan et al. (2022) developed a technique for early-stage detection of breast cancer. Initially, the system is trained using the acquired MRI breast image dataset. Following that, the pre-process function was used to eliminate the training faults. Consequently, the pre-processed data was supplied into the classification layer, and the feature extraction and classification processes were completed. Finally, the proposed model’s findings demonstrate its capability to efficiently detect breast cancer, whether benign or malignant, at an early stage. As a result, the planned mechanism has attained 99.6% more accuracy.

Jabeen et al. (2022) created an approach for classifying breast cancer using US images. The proposed approach deployed a DarkNet-53 deep learning model; the breast US data is first augmented and then retrained. Following that, the pooling layer’s features were recovered, and the best feature was selected using two different optimization strategies. The selected attributes are then fused using a specific approach, and the classification was performed using machine learning techniques. The suggested technique (using feature fusion and Clustered Support Vector Machines(CSVM) classifier) achieved an accuracy of 99.1%. Zerouaoui & Idri (2022) investigated 28 hybrid architectures for breast cancer imaging classification using two datasets (BreakHis and FNAC). They used different classifiers such as MLP, SVM, DT, and KNN, besides seven Deep Learning techniques for feature extraction. The DenseNet201 was utilized by the three top-ranked hybrid architectures that significantly outperformed other hybrid architectures. Using MLP, SVM, and KNN classifiers, they attained average accuracies of 93.85%, 93.21%, and 83.87%, respectively. Chowdhury et al. (2022) developed a breast cancer classification system that uses pretrained transfer learning models to extract fine-tuned characteristics prior to training on histopathological images. It assists users in classifying tissues by allowing them to upload a single histopathological image at a time. The proposed model has achieved an accuracy of 99.58%.

Mohamed et al. (2022) suggested a three-stage, fully automated breast cancer detection method. First, the thermal images are decreased in size to speed up processing. Second, the region of interest is retrieved automatically using the U-Net architecture. Third, they proposed CNN architecture to classify breast tissues. Their model achieved a classification accuracy of 99.33%. Lahoura et al. (2021) established a breast cancer detection system based on cloud computing and Extreme Learning Machine (ELM) as a classifier. They used the WBCD dataset to test multiple classifiers for breast cancer diagnosis. First, the gain ratio approach was utilized to choose the most relevant features and reject extraneous information. Second, numerous cutting-edge algorithms were implemented and compared to ELM on the standalone system. Furthermore, the ELM model was implemented on the Amazon EC2 cloud platform. The experimental results proved an obtained accuracy of 98.68%.

Senan et al. (2021) classified breast cancer based on transfer learning and histopathology images. They created a network based on the AlexNet architecture. Transfer learning was used to provide efficient and accurate classification. ImageNet information can be transferred *via* the network as convolutional features for image histology problem classification. Despite the minimal number of images in the target data set (BreaKHis), they achieved 95% accuracy. Boumaraf et al. (2021) applied the transfer learning technique to the VGG-19 architecture. It evolved into a block-wise fine-tuned architecture on histopathology pictures after pre-training on ImageNet. Finally, they assessed the proposed approach for magnification-dependent breast cancer classification using the BreaKHis dataset. The best-obtained classification accuracies vary from 94.05% to 98.13% utilizing the DL technique for binary classification.

## Methodology

**Figure 2:**
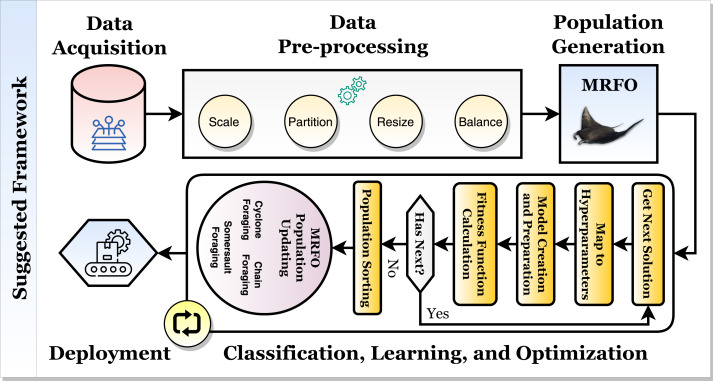
The proposed hybrid breast cancer recognition framework.

Based on histopathological and ultrasound findings, the current study describes a framework for classifying breast cancer automatically and accurately with the help of convolutional neural networks, transfer learning, and the Manta Ray Foraging Optimization for parameters and hyperparameter optimization. The proposed hybrid framework is depicted in Fig. 2.

Figure 2 depicts the framework, which comprises two mechanisms. The model is optimized and created with high state-of-the-art (SOTA) performance metrics in the first learning and optimization mechanism. It consists of five phases: data acquisition, data pre-processing, data splitting, classification and optimization, and production preparation. The second mechanism is the production mechanism. In it, the patient will perform an X-ray scan on the brain, and the scan will be classified using the first suggested classifier. It should be diagnosed with one of “Healthy” or “Tumor”. If the scan is “Healthy,” the patient has a healthy brain. If the scan is “Tumor”, then the patient will perform an MRI scan, which will be classified using the second suggested classifier to determine the tumor (*i.e.,* cancer) type. The tumor can be “Meningioma”, “Glioma”, or “Pituitary”. The following subsections will discuss the inner details of each phase in the first mechanism.

### Phase 1: dataset acquisition

The datasets used in this study were obtained from two publicly available datasets on Kaggle. In summary, the current study depends on two different types of modalities. The first is the histopathological slides, while the second is the ultrasound records. The first dataset, Breast Cancer Dataset (BreaKHis) (Elmasry, 2021), is partitioned into two classes: “Benign” and “Malignant” where each category included 2,479 and 5,304 images respectively. The second dataset, US images data (Fouad, 2021), is divided into three categories: “Benign,” “Malignant,” and “Normal,” with 437, 210, and 133 images in each. The “Normal” class includes cases where there is no tumor while the “Benign” class includes cases where there is a tumor but is considered benign.

In both datasets, data augmentation is used prior to training to up-sample and equalize the number of images per category. After equalization, the first dataset contained 10, 608 images, with each class containing 5, 304 images. Besides, the second dataset contained 1, 311 images, with 437 images in each class. The specifications of the datasets used are summarized in Table 1.

**Table 1:** The used datasets specifications summarization.

**Dataset**	**No. of classes**	**Classes**	**No. of images (Before)**	**No. of images (After)**
**Breast Cancer Dataset (BreaKHis)** Elmasry (2021)	2	“Benign” and “Malignant”	7, 783	10, 608
**Breast Ultrasound data** Fouad (2021)	3	“Benign”, “Malignant”, and “Normal”	780	1, 311

### Phase 2: dataset pre-processing

The second phase pre-processes the datasets by applying four techniques. They are image resizing, dimensional scaling, and data balancing.

**Image Resizing**: The used images are not equal in their dimensions. As a result, in the RGB mode, a bicubic interpolation resizing technique should be used to the size of (128 × 128 × 3).

**Dimensional Scaling**: It utilizes four different scaling techniques, namely (1) normalization, (2) standardization, (3) min-max scaling, and (4) max-abs scaling. The equations underlying them are shown in Eqs. (1)–(4) respectively where *X* represents RGB image, *X*_*output*_ is the output scaled image, µis the image’s mean, *σ* is the standard deviation of the image.

(1) \begin{eqnarray*}{X}_{output}= \frac{X}{\max \nolimits (X)} \end{eqnarray*}

(2) \begin{eqnarray*}{X}_{output}= \frac{X-\mu }{\sigma } \end{eqnarray*}

(3) \begin{eqnarray*}{X}_{output}= \frac{X-\min \nolimits (X)}{\max \nolimits (X)-\min \nolimits (X)} \end{eqnarray*}

(4) \begin{eqnarray*}{X}_{output}= \frac{X}{{|}\max \nolimits (X){|}} \end{eqnarray*}

**Dataset Balancing**: The dataset is imbalanced, which can enhance the misclassification or overfitting in the training and optimization process. To eliminate this issue, a data balancing technique should be utilized. The current study employs data augmentation techniques. They use shifting (width and height), shearing, rotation, flipping (horizontal and vertical axes), zooming, and brightness changes. Table 2 depicts the different utilized data augmentation techniques and their configurations.

**Data Splitting**: There are three subsets in the dataset: training, testing, and validation. The current study employs an 85% to 15% ratio. Initially, the entire dataset is divided into training and testing subsets based on the defined ratio. Finally, the training subset is divided into training and validation subsets based on the same defined ratio.

### Phase 3: classification and optimization

**Figure 3:**
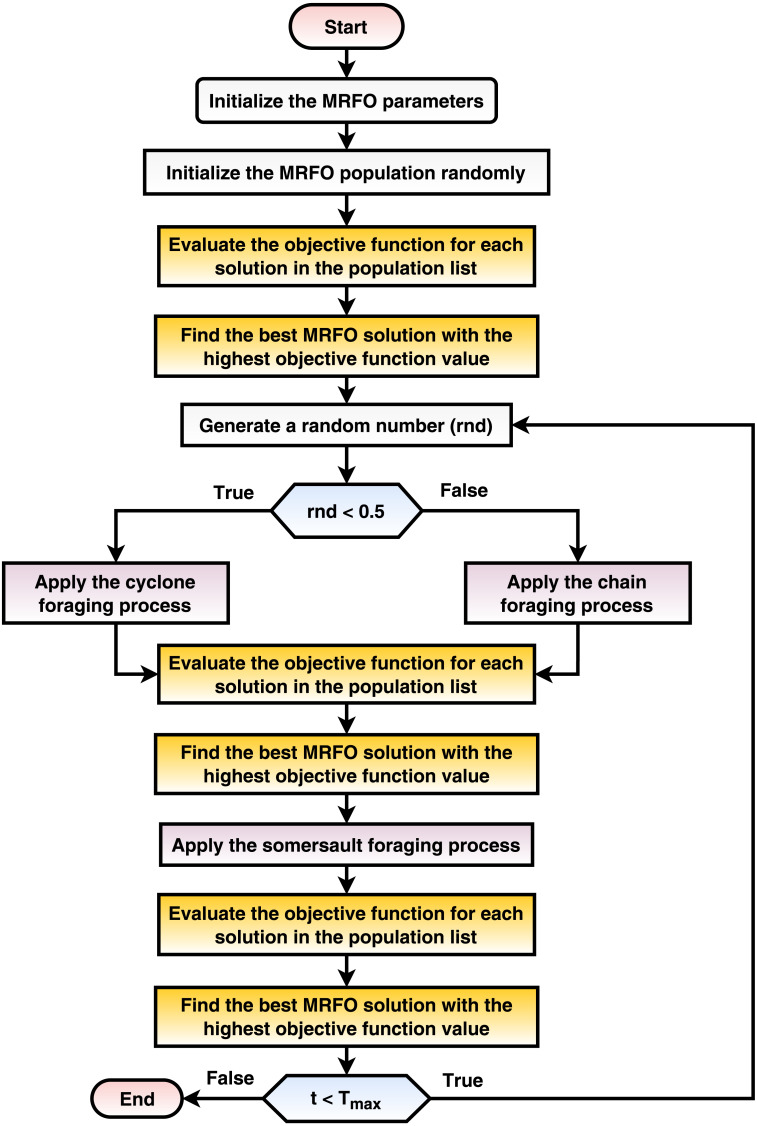
A flowchart summarization of the MRFO steps.

The current phase optimizes the various transfer learning hyperparameters using the MRFO metaheuristic population-based optimizer (*e.g.*, dropout ratio, data augmentation appliance, and scaling technique). The proposed mechanism seeks the best configurations for each pre-trained transfer learning CNN model. This phase implements three internal processes and repeatedly runs for a number of iterations equals *T*_*max*_. The processes are: (1) randomly create the initial population, (2) calculate the fitness function for each solution in the population sack, and (3) update the population concerning the MRFO equations. The process is summarized in Fig. 3 and discussed in detail in the current subsection.

**Table 2:** The different implemented data augmentation techniques and the corresponding configurations.

**Augmentation technique**	**Value**
Rotation	30°
Shear ratio	20%
Zoom ratio	20%
Width shift ratio	20%
Height shift ratio	20%
Brightness change	[0.8:1.2]
Vertical flip	✓
Horizontal flip	✓

Initially, the MRFO population sack is randomly created once at the beginning of the classification and optimization phase. The number of solutions is *N*_*max*_ and set to 10 in the current study. Each solution from the population sack is a vector with a size of (1 × *D*) where each element ∈[0, 1]. Each cell in a solution reflects a specific learning hyperparameter (*e.g.*, dropout ratio and batch size). The current study targets to improve 15 hyperparameters as shown in Table 3. The solution indexing (starting from 1 to 15) and the corresponding hyperparameters definitions and ranges are presented in it. If data augmentation is applied, *D* = 15 and if not, *D* = 7.

**Table 3:** The solution indexing and the corresponding hyperparameters definitions and ranges.

**Element index**	**Corresponding hyperparameter definition**	**Corresponding range**
1	Training loss function	Categorical Crossentropy, Categorical Hinge, KLDivergence, Poisson, Squared Hinge, and Hinge
2	Training batch size	4 → 48 (step = 4)
3	Model dropout ratio	[0 → 0.6]
4	Transfer learning freezing ratio	1 → 100 (step = 1)
5	Weights (i.e., parameters) optimizer	Adam, NAdam, AdaGrad, AdaDelta, AdaMax, RMSProp, SGD, Ftrl, SGD Nesterov, RMSProp Centered, and Adam AMSGrad
6	Dimension scaling technique	Normalize, Standard, Min Max, and Max Abs
7	Utilize data augmentation techniques or not	[*Yes*, *No*]
8	The value of rotation (In the case of data augmentation).	0° → 45° (step = 1°)
9	In the case of data augmentation, width shift value.	[0 → 0.25]
10	The value of height shift if data augmentation is applied	[0 → 0.25]
11	Value of shear in case of data augmentation	[0 → 0.25]
12	Value of Zoom (if data augmentation is used)	[0 → 0.25]
13	Flag for horizontal flipping (if data augmentation is utilized)	[*Yes*, *No*]
14	(If augmentation of data has been applied), the value of Vertical flipping flag	[*Yes*, *No*]
15	Range of brightness changes (if data augmentation is applied)	[0.5 → 2.0]

After that, the fitness (*i.e.,* objective) function score of each solution in the population sack is calculated. The objective function consists of three inner steps: (1) hyperparameters mapping, (2) model creation and preparation, and (3) model training and evaluation. The hyperparameters mapping step maps the solution into the corresponding actual hyperparameters as defined in Table 3. How does the hyperparameters mapping happen internally? A simple calculation is utilized to map the random numbers to the values of the corresponding hyperparameters. This can be done using Eq. (5). For example, if it is required to convert the model dropout ratio (*i.e.,* the third element) from the solution numeric value to the corresponding hyperparameter. The range of the model dropout ratios to select from should be determined first (the current study uses the “ [0 → 0.6]” range). Then, from Eq. (5), if the random numeric value is 0.85 and we have a range from 0 to 0.6, then the value is 0 + 0.85 × (0.6 − 0) = 0.51.

(5) \begin{eqnarray*}\text{Value}=\text{Lower Bound}+\text{Solution}[\text{Element Index}]\times (\text{Upper Bound}-\text{Lower Bound}).\end{eqnarray*}

The target pre-trained transfer learning model is created and compiled with these mapped hyperparameters after mapping each element in the solution to the corresponding hyperparameters. The current study uses the MobileNet, MobileNetV2, MobileNetV3Large, VGG16, VGG19, Xception, DenseNet201, and NASNetMobile pre-trained transfer learning CNN models. Their initial weights (*i.e.,* parameters) are set with the “ImageNet” pre-trained weights. The pre-trained transfer learning model will start the training process for a number of epochs set to 5 in the current study. After the learning (*i.e.,* training), the pre-trained transfer learning model is evaluated on the whole dataset for validation and generalization purposes. The model’s performance is assessed based on different metrics (*e.g.*, precision, accuracy, recall, and F1-score). This study uses a variety of performance metrics illustrated in Eqs. (6) to (11).

(6) \begin{eqnarray*}\text{Accuracy (ACC)}= \frac{TP+TN}{TP+TN+FP+FN} \end{eqnarray*}

(7) \begin{eqnarray*}\text{Precision (P)}= \frac{TP}{TP+FP} \end{eqnarray*}

(8) \begin{eqnarray*}\text{Specificity (S)}= \frac{TN}{TN+FP} \end{eqnarray*}

(9) \begin{eqnarray*}\text{Recall (R)}=\text{Sensitivity}= \frac{TP}{TP+FN} \end{eqnarray*}

(10) \begin{eqnarray*}\text{Dice Coef (DC).}= \frac{2\times TP}{2\times TP+FP+FN} \end{eqnarray*}

(11) \begin{eqnarray*}\text{F1-score}= \frac{2\times \text{Precision}\times \text{Recall}}{\text{Precision}+\text{Recall}} .\end{eqnarray*}

*TP* represents the true positive, *TN* indicates the true negative, *FN* represents the false negative, and *FP* indicates the false positive. After calculating the performance metrics for each solution, the population solutions are sorted in descending order concerning the objective function score. In other words, the best solution is located at the top to determine *X*_*best*_, which is used throughout the process. After that, the population is updated using the MRFO equations. The MRFO works on two branches: cyclone foraging (Eq. (12)) and chain foraging (Eq. (13)). To determine which path to follow, a random value (∈[0, 1]) is generated, and the first branch is followed if the generated random value <0.5 and the second branch is followed otherwise. After updating the solution using either path, each solution’s fitness function is reevaluated again, and *X*_*best*_ is redetermined. After that, somersault foraging (Eq. (14)) is performed. Again, each solution’s fitness function is reevaluated, and *X*_*best*_ is redetermined.

(12) \begin{eqnarray*}{X}_{i}(t+1)= \left\{ \begin{array}{@{}ll@{}} \displaystyle {X}_{rand}+r\times \left( {X}_{rand}-{X}_{i}(t) \right) +\beta \times \left( {X}_{rand}-{X}_{i}(t) \right) , &\displaystyle \text{if}(( \frac{t}{{T}_{max}} \lt rand)~\mathrm{{\XMLAMP}}~(i=1))\\ \displaystyle {X}_{rand}+r\times \left( {X}_{i-1}(t)-{X}_{i}(t) \right) +\beta \times \left( {X}_{rand}-{X}_{i}(t) \right) , &\displaystyle \text{if}(( \frac{t}{{T}_{max}} \lt rand)~\mathrm{{\XMLAMP}}~(i\gt 1))\\ \displaystyle {X}_{best}+r\times \left( {X}_{best}-{X}_{i}(t) \right) +\beta \times \left( {X}_{best}-{X}_{i}(t) \right) , &\displaystyle \text{if}(( \frac{t}{{T}_{max}} \geq rand)~\mathrm{{\XMLAMP}}~(i=1))\\ \displaystyle {X}_{best}+r\times \left( {X}_{i-1}(t)-{X}_{i}(t) \right) +\beta \times \left( {X}_{best}-{X}_{i}(t) \right) , &\displaystyle \text{if}(( \frac{t}{{T}_{max}} \geq rand)~\mathrm{{\XMLAMP}}~(i\gt 1))\\ \displaystyle \end{array} \right. \end{eqnarray*}

(13) \begin{eqnarray*}{X}_{i}(t+1)= \left\{ \begin{array}{@{}ll@{}} \displaystyle {X}_{i}(t)+r\times \left( {X}_{best}-{X}_{i}(t) \right) +\alpha \times \left( {X}_{best}-{X}_{i}(t) \right) , &\displaystyle \text{if}(i=1)\\ \displaystyle {X}_{i}(t)+r\times \left( {X}_{i-1}(t)-{X}_{i}(t) \right) +\alpha \times \left( {X}_{best}-{X}_{i}(t) \right) , &\displaystyle \text{Otherwise}\\ \displaystyle \end{array} \right. \end{eqnarray*}

(14) \begin{eqnarray*}{X}_{i}(t+1)={X}_{i}(t)+S\times \left( {r}_{2}\times {X}_{best}-{r}_{3}\times {X}_{i}(t) \right) \end{eqnarray*}

where *X*_*i*_(*t*) is the *i*th solution at iteration *t*, *t* is the current iteration number, *r* is a random number ∈[0, 1], *β* and *α* are weight coefficients, and *S* is the somersault factor, *r*_1_ is a random number ∈[0, 1], and *r*_2_ is a random number ∈[0, 1]. Algorithm 1 explains how the population (that is, solutions) is updated using MRFO metaheuristic optimization.

 
 
 Algorithm 1: The population (i.e.,  solutions) updating process using the MRFO 
  metaheuristic optimizer                                                                                 ____ 
  1  Function UpdateMRFOSolu- 
     tions(solutions,scoresList,model,trainX,trainY,validationX,validationY,testX,testY ) 
      // Sort the population scores. 
  2  SortMRFOSolutions(solutions,scoresList)                       // Sort the scores list in descending order. 
  3  bestSolution,bestScore = ExtractFromSolutions(solutions,scoresList)    // Extract the best solution 
      and score. 
      // Start the updating process using MRFO equations. 
  4  i = 1                                                              // Initialize the manta-ray’ counter where i ≤ Nmax. 
  5  while (i ≤ Nmax) do 
       6    if (rand < 0.5) then 
        // The cyclone foraging process (Equation 12). 
7    if (t/Tmax < rand) then 
8    randSolution = xL + rand × (xU − xL) 
9    if (i == 1) then 
   // Apply this equation if the current solution is the first one. 
10    solutions[i] = 
    randSolution + r × (randSolution − solutions[i]) + β × (randSolution − solutions[i]) 
11    else 
   // Apply this equation if the current solution is not the first one. 
12    solutions[i] = 
    randSolution+r×(solutions[i − 1] − solutions[i])+β×(randSolution − solutions[i]) 
13    else 
14    if (i == 1) then 
   // Apply this equation if the current solution is the first one. 
15    solutions[i] = 
    bestSolution + r × (bestSolution − solutions[i]) + β × (bestSolution − solutions[i]) 
16    else 
   // Apply this equation if the current solution is not the first one. 
17    solutions[i] = 
    bestSolution + r × (solutions[i − 1] − solutions[i]) + β × (bestSolution − solutions[i]) 
18    else 
        // The chain foraging process (Equation 13.) 
19    if (i == 1) then 
   // Apply this equation if the current solution is the first one. 
20    solutions[i] = 
    solutions[i] + r × (bestSolution − solutions[i]) + α × (bestSolution − solutions[i]) 
21    else 
   // Apply this equation if the current solution is not the first one. 
22    solutions[i] = 
    solutions[i] + r × (solutions[i − 1] − solutions[i]) + α × (bestSolution − solutions[i]) 
23    i = i + 1                                                                                        // Increment the counter. 
 24  i = 1                                                              // Initialize the manta-ray’ counter where i ≤ Nmax. 
 25  newScoresList = []                                                                              // Initialize the scores list.  
 26  while (i ≤ Nmax) do 
       27    score = CalculateFitnessScore (model,solutions[i],trainX,trainY,validationX,validationY ) 
    // Calculate the fitness score (i.e., accuracy) for the current solution. 
28    if (score > bestScore) then 
     29    bestScore = score 
30    bestSolution = solutions[i] 
31    i = i + 1                                                                         // Increment the manta-ray’ counter. 
      // The somersault foraging process (Equation 14). 
 32  i = 1                                                              // Initialize the manta-ray’ counter where i ≤ Nmax. 
 33  while (i ≤ Nmax) do 
       34    solutions[i] = solutions[i] + S × (r2 × bestSolution − r3 × solutions[i]) i = i + 1    // Increment the 
    counter. 
 35  i = 1                                                              // Initialize the manta-ray’ counter where i ≤ Nmax. 
 36  newScoresList = []                                                                              // Initialize the scores list. 
 37  while (i ≤ Nmax) do 
       38    score = CalculateFitnessScore (model,solutions[i],trainX,trainY,validationX,validationY ) 
    // Calculate the fitness score (i.e., accuracy) for the current solution. 
39    if (score > bestScore) then 
     40    bestScore = score 
41    bestSolution = solutions[i] 
42    i = i + 1                                                                         // Increment the manta-ray’ counter. 
 43  return solutions                                                           // Return the updated population 
 44  End Function    

### The proposed framework steps

Iteratively, the steps are computed over a maximum number of iterations *T*_*max*_. The optimization iterations used the MRFO; the best possible model combination can then be applied to any further analysis, such as the production mechanism as prescribed in the framework. Finally, algorithm 2 summarizes parameter learning and hyperparameter optimization.


 
 
  Algorithm 2: The suggested framework pesudocode                                          ____ 
    Input: model, dataset                                 // The required model name and the dataset records 
      Output: best,bestScore                                               // The best overall score and solution 
  1  trainX,validationX,testX,trainY,validationY,testY = SplitDataset (dataset)          // Partition the 
      dataset into training, testing, and validation portions concerning the defined split ration. 
  2  model = CreateTLPretrainedModel()    // Create the initial transfer learning pre-trained CNN model. 
  3  solutions = GenerateInitialSolutions()                            // Generate the initial solutions randomly. 
      // Execute the learning MRFO hyperparameters optimization process for Tmax iterations. 
  4  t = 1                                                                // Initialize the iterations’ counter where t ≤ Tmax. 
  5  while (t ≤ Tmax) do 
          // Calculate the scores for each solution in the population sack. 
6    i = 1                                                            // Initialize the MRFO’s counter where i ≤ Nmax. 
7    scoresList = []                                                                              // Initialize the scores list. 
8    while (i ≤ Nmax) do 
     9    score = 
    CalculateFitnessScore (model,solutions[i],trainX,trainY,validationX,validationY,testX,testY ) 
    // Calculate the objective function score (i.e., accuracy) for the current solution. 
10    Append(score,scoresList)                                    // Calculate the score into the scores list. 
11    i = i + 1                                                                      // Increment the MRFO’s counter. 
    // Update the population using MRFO (Algorithm 1). 
12    solutions = 
    UpdateMRFOSolutions(solutions,scoresList,model,trainX,trainY,validationX,validationY,testX,testY ) 
13    t = t + 1                                                                          // Increment the iterations counter. 
 14   best,bestScore                                     // Return the best score and the corresponding solution    


## Numerical results and analysis

This section presents the test results for various applied experiments. Furthermore, it provides comments on the reported results. A summary of all experiment configurations is provided in Table 4.

**Table 4:** Common experiments configurations.

**Configuration**	**Specifications**
Datasets	Table 1
Apply dataset shuffling?	Yes (Random)
Input image size	(128 × 128 × 3)
Hyperparameters metaheuristic optimizer	Manta-ray foraging algorithm (MRFO)
Train split ratio	85% to 15% (i.e., 85% for training (and validation) and 15% for testing)
MRFO population size	10
MRFO iterations count	10
Epochs number	5
O/P activation function	SoftMax
Pre-trained models	InceptionV3, Xception, EfficientNetB7, NASNetLarge, VGG19, SeNet154, DenseNet201, and ResNet152V2
Pre-trained parameters initializers	ImageNet
Hyperparameters	Table 3
Scripting language	Python
Python major packages	Tensorflow, Keras, NumPy, OpenCV, and Matplotlib
Working environment	Google Colab with GPU (i.e., Intel(R) Xeon(R) CPU @ 2.00 GHz, Tesla T4 16 GB GPU, CUDA v.11.2, and 12 GB RAM)

### Experiments with the binary dataset

Table 5 summarizes the configurations of the binary dataset. Table 6 displays the confusion matrix results concerning the binary dataset and the best solutions for each pre-trained model after learning and optimizing the model. Based on these results, the Xception model represents the lowest FP and FN values. However, EfficientNetB7 has the highest values for FP and FN.

**Table 5:** Binary dataset specific experiments configurations.

**Configuration**	**Specifications**
Dataset source	https://www.kaggle.com/anaselmasry/breast-cancer-datasetElmasry (2021)
Number of classes	2
Classes	(‘Benign’ and ‘Malignant’)
Dataset size before data balancing	“Benign”: 2,479 and “Malignant”: 5,304
Dataset size after data balancing	“Benign”: 5,304 and “Malignant”: 5,304

**Table 6:** Confusion matrix results concerning the binary dataset.

**Model name**	**TP**	**TN**	**FP**	**FN**
InceptionV3	10,288	10,288	320	320
Xception	10,363	10,363	241	241
EfficientNetB7	7,916	7,916	2,668	2,668
NASNetLarge	9,817	9,817	791	791
VGG19	10,308	10,308	300	300
SeNet154	10,171	10,171	413	413
DenseNet201	10,350	10,350	242	242
ResNet152V2	10,225	10,225	383	383

A list of the best solution combinations is presented in Table 7. According to the analysis, five different models recommend using the KLDivergence loss. Moreover, the Nesterov-based models are recommended by three models. Three models recommend the min-max scalers. Different performance metrics can be derived by combining the values reported in Table 6 and the learning history. There are two types of metrics presented.

**Table 7:** Learning and optimization best solutions concerning the binary dataset.

**Model name**	**InceptionV3**	**Xception**	**EfficientNetB7**	**NASNetLarge**	**VGG19**	**SeNet154**	**DenseNet201**	**ResNet152V2**
**Loss**	KLDivergence	KLDivergence	Poisson	KLDivergence	Poisson	KLDivergence	Categorical Crossentropy	KLDivergence
**Batch size**	12	44	28	12	12	36	32	48
**Dropout**	0.41	0.28	0.25	0.33	0.47	0.08	0.16	0.43
**TL learn ratio**	91	56	92	70	33	79	49	62
**Optimizer**	AdaGrad	AdaMax	SGD Nesterov	SGD	SGD	SGD Nesterov	AdaGrad	SGD Nesterov
**Scaler**	Normalization	Normalization	MinMax	Normalization	Standardization	MinMax	MaxAbs	MinMax
**Apply augmentation**	Yes	Yes	No	No	No	Yes	Yes	No
**Rotation range**	1	26	N/A	N/A	N/A	11	24	N/A
**Width shift range**	0.22	0.17	N/A	N/A	N/A	0.18	0.03	N/A
**Height shift range**	0.22	0.25	N/A	N/A	N/A	0.06	0.03	N/A
**Shear range**	0.25	0.08	N/A	N/A	N/A	0.14	0.12	N/A
**Zoom range**	0.24	0.13	N/A	N/A	N/A	0.16	0.17	N/A
**Horizontal flip**	Yes	No	N/A	N/A	N/A	No	Yes	N/A
**Vertical flip**	Yes	Yes	N/A	N/A	N/A	No	No	N/A
**Brightness range**	1.18–1.24	1.28–1.32	N/A	N/A	N/A	1.64–1.78	0.65–1.72	N/A

The first set of metrics represents those that need to be maximized (*e.g.*, Acc, F1-score, P, Sensitivity, Recall, S, AUC, IOU, Dice, and Cosine Similarity). The second one indicates the metrics that must be minimized (*e.g.*, Logcosh Error, Mean Absolute Error, Mean Squared Error, Mean Squared Logarithmic Error, and Root Mean Squared Error). Table 8 reports category metrics for the first category while Table 9 reports metrics for the second category. It is obvious that the Xception pre-trained model is superior to others when it comes to the classification of two-classes datasets. There is no difference between Recall and Sensitivity regarding results or formulas.

**Table 8:** The binary dataset experiments with the maxmimized metrics.

**Model name**	**Accuracy**	**F1**	**Precision**	**Sensitivity**	**Recall**	**Specificity**	**AUC**	**IoU**	**Dice**	**Cosine similarity**
InceptionV3	96.98%	96.98%	96.98%	96.98%	96.98%	96.98%	99.14%	97.07%	97.47%	97.41%
Xception	97.73%	97.73%	97.73%	97.73%	97.73%	97.73%	99.50%	97.90%	98.17%	98.04%
EfficientNetB7	74.79%	74.79%	74.79%	74.79%	74.79%	74.79%	81.85%	74.95%	78.83%	80.26%
NASNetLarge	92.54%	92.54%	92.54%	92.54%	92.54%	92.54%	98.00%	91.55%	93.01%	94.02%
VGG19	97.17%	97.17%	97.17%	97.17%	97.17%	97.17%	99.65%	95.53%	96.45%	97.57%
SeNet154	96.10%	96.10%	96.10%	96.10%	96.10%	96.10%	99.03%	95.17%	96.04%	96.80%
DenseNet201	97.72%	97.72%	97.72%	97.72%	97.72%	97.72%	99.57%	89.33%	92.02%	96.98%
ResNet152V2	96.39%	96.39%	96.39%	96.39%	96.39%	96.39%	99.19%	94.92%	95.90%	96.91%

**Table 9:** The binary dataset experiments with the minimized metrics.

**Model name**	**Logcosh error**	**Mean absolute error**	**Mean squared error**	**Mean squared logarithmic error**	**Root mean squared error**
InceptionV3	0.011	0.038	0.024	0.012	0.156
Xception	0.008	0.027	0.018	0.009	0.135
EfficientNetB7	0.082	0.318	0.177	0.087	0.421
NASNetLarge	0.025	0.105	0.054	0.027	0.233
VGG19	0.010	0.053	0.022	0.011	0.150
SeNet154	0.014	0.059	0.030	0.015	0.172
DenseNet201	0.015	0.120	0.031	0.016	0.177
ResNet152V2	0.013	0.062	0.029	0.014	0.169

### Three-classes dataset experiments

The three-class dataset is summarized in Table 10. According to Table 11, the TP, TN, FP, and FN of the best solutions are available after learning and optimizing each pre-trained model. According to the results, the ResNet152V2 pre-trained model has the lowest FP and FN values. Conversely, EfficientNetB7 has the highest FP and FN values. Table 12 shows the optimal combination of each model. This study shows that five models recommend Categorical Cross entropy Loss. Three models recommend standardization, and four recommend normalization scalers. Three models recommend Adam as an optimizer. Seven models recommended performing data augmentation, with six recommending vertical flipping and five recommending horizontal flipping.

**Table 10:** Three-classes specific experiments configurations.

**Configuration**	**Specifications**
Dataset source	https://www.kaggle.com/ahmedfouad55/breast-ultrasound-dataFouad (2021)
Number of classes	3
Classes	(‘Benign’, ‘Malignant’, and ‘Normal’)
Dataset size before data balancing	“Benign”: 437, “Malignant”: 210, and “Normal”: 133
Dataset size after data balancing	“Benign”: 437, “Malignant”: 437, and “Normal”: 437

**Table 11:** Confusion matrix results concerning the three-classes dataset.

**Model name**	**TP**	**TN**	**FP**	**FN**
InceptionV3	1191	2521	55	97
Xception	1267	2584	32	41
EfficientNetB7	875	2475	117	421
NASNetLarge	1184	2575	41	124
VGG19	1053	2471	121	243
SeNet154	1257	2572	36	47
DenseNet201	1204	2491	61	72
ResNet152V2	1294	2603	13	14

**Table 12:** The promising solutions concerning the three-classes dataset.

**Model name**	**InceptionV3**	**Xception**	**EfficientNetB7**	**NASNetLarge**	**VGG19**	**SeNet154**	**DenseNet201**	**ResNet152V2**
**Loss**	KLDivergence	Categorical Crossentropy	KLDivergence	Categorical Crossentropy	Categorical Crossentropy	KLDivergence	Categorical Crossentropy	Categorical Crossentropy
**Batch size**	28	4	24	4	24	8	44	4
**Dropout**	0.25	0	0.38	0	0.06	0.09	0.46	0
**TL learn ratio**	47	0	68	0	40	54	81	0
**Optimizer**	RMSProp	Adam	AdaMax	Adam	AdaGrad	SGD Nesterov	SGD	Adam
**Scaler**	Standardization	Normalization	Standardization	Normalization	Standardization	Normalization	Standardization	Normalization
**Apply augmentation**	Yes	Yes	Yes	Yes	Yes	No	Yes	Yes
**Rotation range**	13	0	6	0	3	N/A	28	0
**Width shift range**	0.12	0	0.22	0	0.04	N/A	0.11	0
**Height shift range**	0.11	0	0.2	0	0.1	N/A	0.16	0
**Shear range**	0.06	0	0.1	0	0.02	N/A	0.13	0
**Zoom range**	0.03	0	0.24	0	0.06	N/A	0.06	0
**Horizontal flip**	No	Yes	Yes	Yes	Yes	N/A	No	Yes
**Vertical flip**	Yes	Yes	No	Yes	Yes	N/A	Yes	Yes
**Brightness range**	1.03–1.07	0.5–0.5	1.35–1.59	0.5–0.5	0.93–0.94	N/A	1.25–1.37	0.5–0.5

Different performance metrics can be derived from the values provided in Table 11 and the learning history. There are two types of metrics reported. The first aspect shows the metrics that need to be maximized (*i.e.,* Acc, F1-score, P, Sensitivity, Recall, S, AUC, IoU, DC, and Cosine Similarity). On the other hand, the second represents the metrics that must be minimized (namely, Logcosh Error, Mean Absolute Error, Mean Squared Error, Mean Squared Logarithmic Error, and Root Mean Squared Error). Table 13 reports on category metrics for the first category, while Table 14 reports on category metrics for the second category. Compared to others, the ResNet152V2 pretrained model is the best when considering the dataset with three classes. Both Recall and Sensitivity reflect the same results and formulas.

**Table 13:** The three-classes dataset experiments with the maxmimized metrics.

**Model name**	**Accuracy**	**F1**	**Precision**	**Sensitivity**	**Recall**	**Specificity**	**AUC**	**IoU**	**Dice**	**Cosine similarity**
InceptionV3	94.41%	93.81%	95.34%	92.47%	92.47%	97.86%	98.80%	88.69%	90.95%	94.71%
Xception	97.09%	97.04%	97.55%	96.87%	96.87%	98.78%	99.03%	93.65%	94.98%	97.06%
EfficientNetB7	79.40%	74.33%	86.43%	67.52%	67.52%	95.49%	92.61%	69.70%	74.64%	81.80%
NASNetLarge	95.11%	92.15%	96.18%	90.52%	90.52%	98.43%	99.29%	87.78%	90.13%	94.30%
VGG19	85.49%	84.70%	89.24%	81.25%	81.25%	95.33%	96.89%	79.44%	83.17%	88.47%
SeNet154	96.93%	96.70%	97.16%	96.40%	96.40%	98.62%	99.48%	92.61%	94.19%	96.76%
DenseNet201	95.06%	94.73%	95.13%	94.36%	94.36%	97.61%	98.84%	94.31%	95.20%	95.76%
ResNet152V2	99.01%	98.96%	99.01%	98.93%	98.93%	99.50%	99.77%	98.28%	98.60%	98.97%

**Table 14:** The three-classes dataset experiments with the minimized metrics.

**Model name**	**Logcosh error**	**Mean absolute error**	**Mean squared error**	**Mean squared logarithmic error**	**Root mean squared error**
InceptionV3	0.016	0.090	0.033	0.016	0.183
Xception	0.009	0.050	0.019	0.009	0.139
EfficientNetB7	0.051	0.254	0.106	0.052	0.326
NASNetLarge	0.017	0.099	0.036	0.018	0.190
VGG19	0.033	0.168	0.069	0.034	0.263
SeNet154	0.010	0.058	0.021	0.010	0.144
DenseNet201	0.012	0.048	0.026	0.013	0.162
ResNet152V2	0.003	0.014	0.007	0.003	0.083

### Graphical summarizations

**Figure 4:**
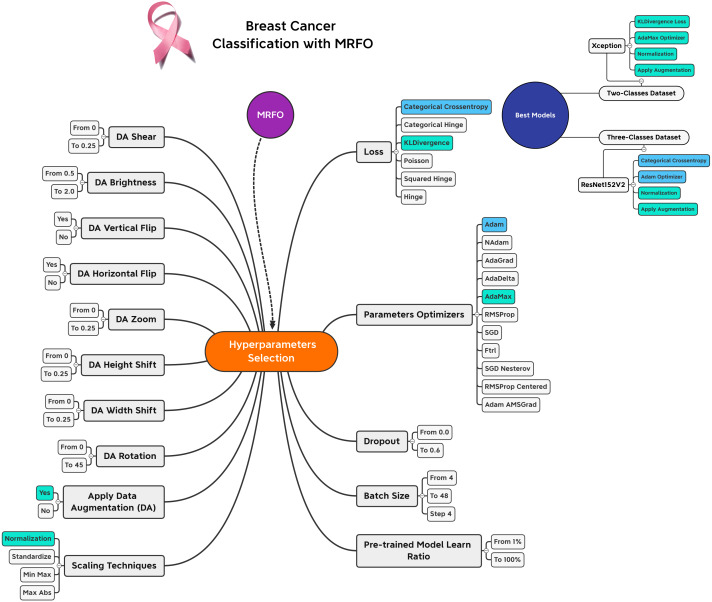
Hyperparameters selection and best combinations graphical summarization.

Based on the experiments conducted on the suggested approach, Fig. 4 shows the best combination of different alternatives.

**Figure 5:**
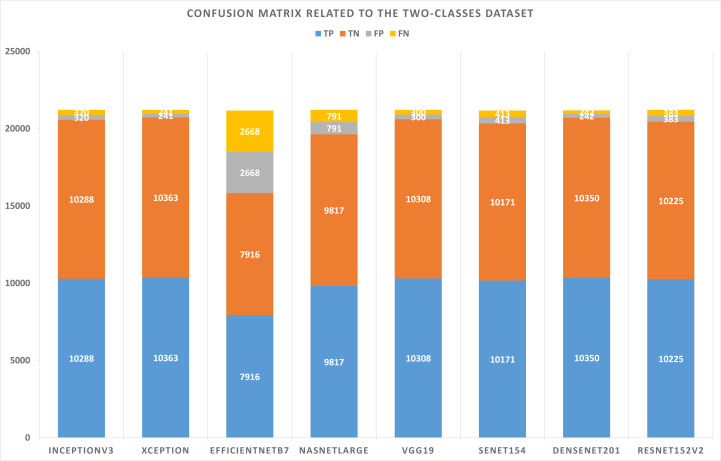
Confusion matrix related to the two-classes dataset.

**Figure 6:**
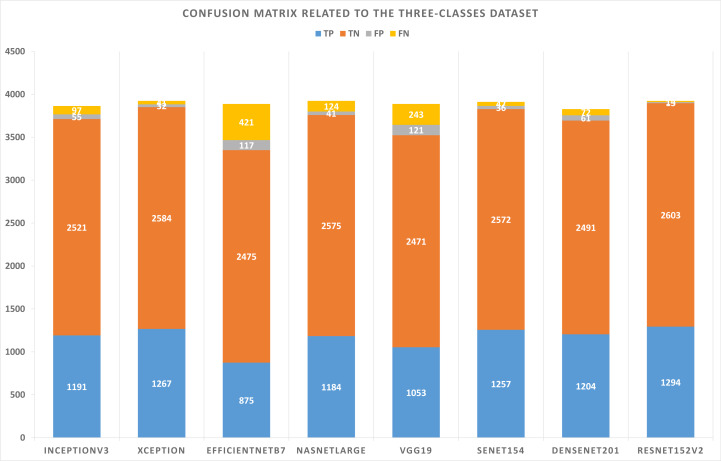
Confusion matrix related to the three-classes dataset.

**Figure 7:**
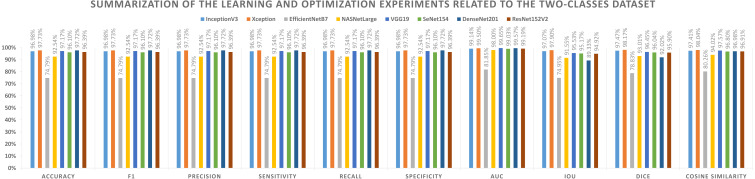
Summarization of the learning and optimization experiments related to the two-classes dataset.

**Figure 8:**
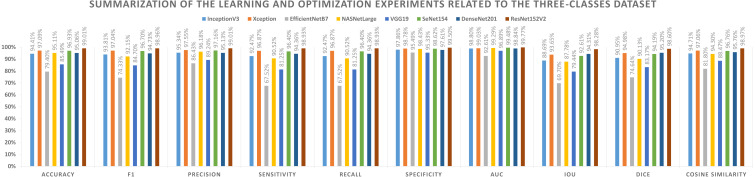
Summarization of the learning and optimization experiments related to the three-classes dataset.

As shown in Figs. 5 and 6, confusion matrices are presented for two-classes and three-class datasets, respectively.

A graphical summary of the reported learning and optimization results is presented in Figs. 7 and 8, respectively, using two- and three-class datasets.

### Related studies comparisons

The proposed approach is compared with other state-of-the-art approaches in Table 15. It compares the whole systems as black boxes. In comparison to related studies, the present study has demonstrated superior results.

## Conclusions

Deep learning has been used to diagnose or detect diseases, much like radiologists do. Another benefit of deep learning would be to double-check the areas radiologists sometimes overlook, thus improving the accuracy of readings. This article presents a new deep convolutional neural network (CNN) approach combining transfer learning with the Manta Ray Foraging Optimization (MRFO) for analyzing histopathological slides and ultrasound images. Optimizing CNN hyperparameters through MRFO increases the performance of the framework. In addition, this approach uses the Manta Ray Foraging Optimization (MRFO) method to drive down the run-time resource requirements of the trained deep learning models, thus improving their adaptability and reducing their run-time costs. Several pre-trained CNNs were used for our experiments, including MobileNet, MobileNetV2, MobileNetV3Large, VGG16, VGG19, Xception, DenseNet201, and NASNetMobile. The datasets used for the study are the Breast Cancer Dataset, which is binary classified, and the Breast Ultrasound Dataset, which is classified into one of the three classes: Benign, Malignant, and Normal. Several pre-training steps are undertaken before the training process begins for each dataset, including data augmentation, image resizing, dimensional scaling, and data balancing. For two-class datasets, the Xception pretrained model is the best. However, when it comes to the three-classes data, the ResNet152V2 pre-trained model is the best. The proposed framework scored 97.73% on the histopathological data and 99.01% on the ultrasound data based on accuracy. In comparison with state-of-the-art techniques, the proposed framework delivered superior results.

**Table 15:** Comparison between the suggested approach and related studies.

**Study**	**Year**	**Dataset**	**Approach**	**Best accuracy**
Wang et al. (2016)	2016	Histopathological	GoogLeNet DL	98.40%
Han et al. (2017b)	2017	Histopathological	Structured DL	93.20%
Han et al. (2017a)	2017	Ultrasound	GoogLeNet DL	90.00%
Jannesari et al. (2018)	2018	Histopathological	DL	96.40%
Golatkar, Anand & Sethi (2018)	2018	Histopathological	DL	93.00%
Fujioka et al. (2019)	2019	Ultrasound	GoogLeNet DL	92.50%
Hijab et al. (2019)	2019	Ultrasound	VGG16 DL	97.00%
Boumaraf et al. (2021)	2021	Histopathological	VGG19 DL	98.13% and 88.95%
Senan et al. (2021)	2021	Histopathological	AlexNet DL	95.00%
Current Study	2022	Histopathological	Hybrid (MRFO and CNN)	97.73%
Current Study	2022	Ultrasound	Hybrid (MRFO and CNN)	99.01%

## Additional Information and Declarations

### Data Availability

The following information was supplied regarding data availability: The data is available at GitHub:
https://github.com/HossamBalaha/Classification-of-breast-cancer-using-a-Manta-ray-foraging-optimized-transfer-learning-Framework.
